# Membranous nephropathy: Systems biology-based novel mechanism and traditional Chinese medicine therapy

**DOI:** 10.3389/fphar.2022.969930

**Published:** 2022-09-13

**Authors:** Hua Miao, Yamei Zhang, Xiaoyong Yu, Liang Zou, Yingyong Zhao

**Affiliations:** ^1^ School of Pharmacy, Zhejiang Chinese Medical University, Hangzhou, Zhejiang, China; ^2^ Key Laboratory of Clinical Genetics & Key Disciplines of Clinical Pharmacy, Affiliated Hospital and Clinical Medical College of Chengdu University, Chengdu, Sichuan, China; ^3^ Department of Nephrology, Shaanxi Traditional Chinese Medicine Hospital, Xi’an, Shaanxi, China; ^4^ School of Food and Bioengineering, Chengdu University, Chengdu, Sichuan, China

**Keywords:** membranous nephropathy, anti-PLA 2 R antibody, gut microbiota, long non-coding RNAs, metabolite biomarkers, traditional Chinese medicine

## Abstract

Membranous nephropathy (MN) is a renal-limited non-inflammatory autoimmune disease in the glomerulus, which is the second or third main cause of end-stage kidney diseases in patients with primary glomerulonephritis. Substantial achievements have increased our understanding of the aetiology and pathogenesis of murine and human MN. The identification of nephritogenic autoantibodies against neutral endopeptidase, phospholipase A_2_ receptor (PLA_2_R) and thrombospondin type-1 domain-containing 7A (THSD7A) antigens provide more specific concept-driven intervention strategies for treatments by specific B cell-targeting monoclonal antibodies to inhibit antibody production and antibody-antigen immune complex deposition. Furthermore, additional antibody specificities for antigens have been discovered, but their pathogenic effects are uncertain. Although anti-PLA_2_R and anti-THSD7A antibodies as a diagnostic marker is widely used in MN patients, many questions including autoimmune response development, antigenic epitopes, and podocyte damage signalling pathways remain unresolved. This review describes the current available evidence regarding both established and novel molecular mechanisms based on systems biology approaches (gut microbiota, long non-coding RNAs, metabolite biomarkers and DNA methylation) in MN, with an emphasis on clinical findings. This review further summarizes the applications of traditional Chinese medicines such as *Tripterygium wilfordii* and *Astragalus membranaceus* for MN treatment. Lastly, this review considers how the identification of novel antibodies/antigens and unresolved questions and future challenges reveal the pathogenesis of MN.

## 1 Introduction

Membranous nephropathy (MN) is a major cause of antibody-associated nephrotic syndrome among adults (Gianassi et al., 2019; Ronco et al., 2021). The development of disease is triggered by accumulation of immune complex deposition in the subepithelial region with local complement activation and injury to podocytes and glomerular basement membrane (GBM) thickening (Feng et al., 2020). While 70%–80% of MN is classified as idiopathic or primary MN, the rest of MN is triggered by secondary causes, such as infections, malignancies or autoimmune diseases (Ronco et al., 2021). The two distinct types require different management approaches. In primary MN, approximately 30% of patients can improve by spontaneous remission, while the remainder show persistent proteinuria or progression to end-stage renal disease (Ronco et al., 2021). In the most severe cases, immunosuppressant treatment is required. Secondary MN requires treatment of the underlying diseases. In most patients, MN is an autoimmune disease mediated by autoantibodies directed against phospholipase A_2_ receptor (PLA_2_R) or, more unusually, thrombospondin type-1 domain-containing 7A (THSD7A) (Deng et al., 2021). However, THSD7A is not specific for primary MN. When secondary causes are excluded, the disease is called primary MN. To date, the diagnosis of MN can only be determined by renal biopsy. In recent years, MN pathogenesis was investigated by using both patients and animal models to help understand the underlying molecular mechanisms for development of immune complex deposition in GBM (Sinico et al., 2016).

This review considers the underlying molecular pathomechanisms of MN, with a particularly focus on novel pathomechanisms such as dysbiosis of gut microbiota, dysregulation of Non-coding RNAs (long non-coding RNAs, microRNAs), aberrant expression of identified proteins by using proteomics and the disorder of endogenous metabolites by metabolomics, as well as altered DNA methylation. These findings will provide strategies for patient diagnosis and monitoring and the possibility for novel treatments. We summarize the animal models of MN that helped to understand human disease. This review further describes the applications of traditional Chinese medicines such as *Tripterygium wilfordii* and *Astragalus membranaceus* for MN treatment.

## 2 The underlying molecular pathomechanisms of IMN in animal models

Three underlying mechanisms elucidated formation of subepithelial complex deposits in both the animal model and human MN (Jiang et al., 2020; Xu et al., 2020). The first mechanism is associated with circulating immune complexes (CIC) deposition described in chronic serum sickness; the second and third underlying mechanisms are associated with *in situ* immune complex formation in the capillary wall of glomerular, where the antigen is either an artificially planted exogenous antigen or a native podocyte antigen bound to the podocyte or basement membrane.

### 2.1 Heymann nephritis rat model

In 1959, Heymann et al. first established a rat MN model, referred to as active Heymann nephritis, which was mediated by immunizing Lewis rats using intrarenal extracts and remind human disease (Heymann et al., 1959). Because subepithelial complex deposits were triggered from intrarenal brush-border membrane rather than glomerular extracts, complex deposits were initially thought to lead to glomerular trapping of CIC containing brush-border-associated antigens and their corresponding antibodies (Sinico et al., 2016; Jiang et al., 2020). However, the rat model of passive Heymann nephritis injected by using the rabbit anti-rat brush-border antibodies argued against a role for CIC (Sinico et al., 2016; Xu et al., 2020). In the passive Heymann nephritis rat model, immune complex deposits occur in minutes after antibody injection, which argues against immune complex formation in circulation followed by their deposition in capillary wall of the glomerulus. Based on *ex vivo* and isolated perfused renal systems, several earlier seminal publications have highlighted that anti-brush-border antibodies could bind to an antigenic target in podocytes (Sinico et al., 2016; Xu et al., 2020), which demonstrated that MN was mediated by *in situ* formation of immune complexes.

**FIGURE 1:**
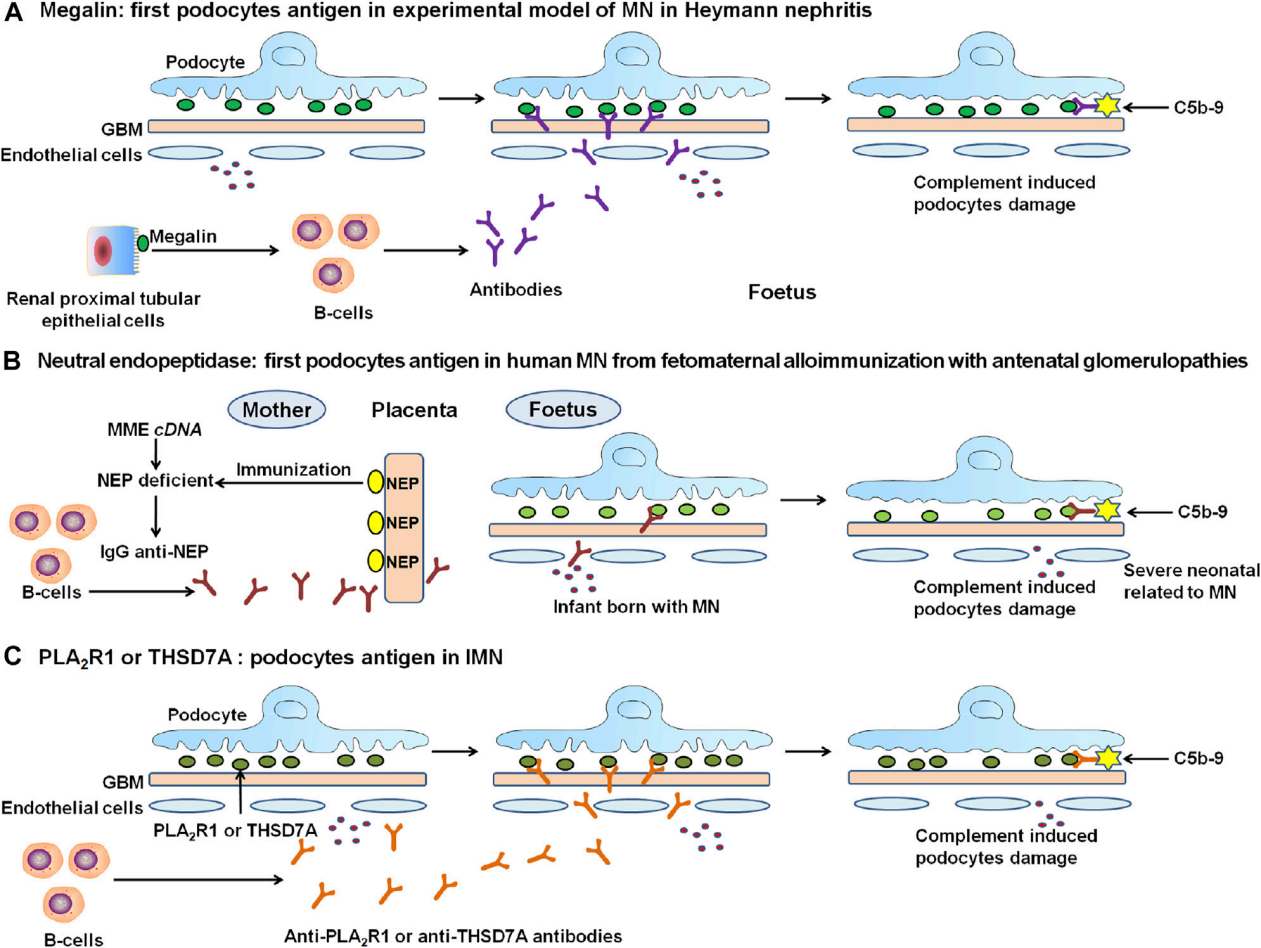
The discovery and proposed mechanisms of the representative antigens in the experimental MN and human IMN. **(A)** In 1982, the polyspecific receptor megalin was identified in Heymann nephritis, but not on human podocytes. **(B)** In humans, progress in MN started in 2002 with the discovery of target antigen namely neutral endopeptidase (NEP) as a targeting antigen in alloimmune neonatal MN. **(C)** In 2009, Novel causal antigen PLA_2_R, which is the first podocyte autoantigen identified in human IMN. Followed by PLA_2_R, in 2014, second antigen THSD7A was identified in human IMN. PLA_2_R-related and THSD7A-related MN accounted for about 70% and 1%–5% of IMN patients, respectively. The formation of immune complexes is the circulation of antibodies binding to endogenous antigens in podocytes. Once formed, these complexes are capped then released into the subepithelial region. They attach to GBM, resist degradation, and persist as immune deposits. Immune deposits form by repeated cycles, which could activate complement pathway. The deposits of subepithelial immune complexes and complement activation damage the podocyte and lead to severe proteinuria.

In the early 1980s, Kerjaschki et al. identified the principal autoantigen megalin (also known as low density lipoprotein receptor-related protein 2), with a molecular weight of ∼600 kDa, in both active and passive Heymann nephritis (Kerjaschki and Farquhar, 1982). Megalin is a large podocyte trans-membrane protein. This polyspecific receptor, as a member of the low-density lipoprotein receptor superfamily, occurs at the sole of podocyte foot processes in the clathrin-coated pits (Figure 1). Immune deposit formation is fuelled by *de novo* synthesis of megalin. Epitope mapping analysis revealed that although subepithelial immune deposits were mediated by 4 megalin ligand-binding domains, full-blown disease with proteinuria was related to a specific epitope in first ligand-binding domain located in a 60 kDa N-terminal fragment (Larsen et al., 2018), and with intramolecular epitope spreading (Zhang et al., 2021).

Heymann nephritis-like MN was also demonstrated in mice and rats (Assmann et al., 1985), where the target antigen was dipeptidyl peptidase 4. The animal model of passive Heymann nephritis was mediated in mice injected by using anti-podocyte serum (Meyer-Schwesinger et al., 2011). Although the findings from Heymann nephritis provided an important contribution to our understanding of the MN underlying pathogenetic mechanisms, there is no evidence that megalin was implicated in human MN.

### 2.2 Cationic bovine serum albumin-Induced IMN rat model

Border and his colleagues established a MN animal model using rabbits immunized with CBSA in 1982 (Border et al., 1982). Their experiments hypothesised that antigen charge was a key mediator for formation of subepithelial deposits, given a negative charge of the capillary wall in the glomeruli. Only rabbits immunized with CBSA showed subepithelial complex deposits of Immunoglobulin G (IgG) and complement C3, whereas those receiving anionic or native (neutral) bovine serum albumin featured mostly mesangial deposits. Those animals immunized with CBSA showed severe proteinuria. The rabbit perfused renal tissues indicated that the complexes were generated *in situ* after CBSA bound to anionic heparin sulphate proteoglycans in GBM, followed by antibody binding (Adler et al., 1983). bovine serum albumin chemical cationization changed its immunogenicity and caused antibody formation of low avidity and precipitability, thus promoting the complex dissociation and access of free CBSA to rabbit the capillary wall in the glomerulus (Koyama et al., 1986; Bass et al., 1990). This model was also reported in other mammal animals such as mice, rats, and dogs (Jiang et al., 2020; Sun et al., 2020). Except for CBSA, other potential antigens can also implant in the capillary wall from the glomerulus, including enzymatically modified nucleosomes that contain cationic histones and may play a role in lupus-related MN, and lectin or cationized ferritin (Beck and Salant, 2014; Liu et al., 2019).

### 2.3 Anti-podocyte nephritis mouse model

The CIC deposition in tissues is a well-known characteristic of chronic serum sickness, which is treated by the administration of exogenous antigen at a constant dose. In glomeruli, CIC location is strongly dependent on antibody response magnitude, immune complex size, antigen characteristics including charge, size, hemodynamics, and pharmacological factors (Couser and Salant, 1980; Alexander et al., 2016). If antigen dose is changed based on the circulating antibody response, to keep up a chronic condition of antigen excess, deposits are nearly exclusively subepithelial regardless of the concentration of antibody response (Zhu, 2016). Therefore, it is speculated that small CIC is made up of low-avidity antibody and oligovalent antigen might traverse the capillary wall.

### 2.4 PLA_2_R1-dependent mouse model

PLA_2_R1 is the main autoantigen in MN patients. Meyer-Schwesinger and colleagues established a novel PLA_2_R1-dependent mouse model that was produced using a transgenic mouse line that expressed full-length PLA_2_R1 in murine podocytes (Meyer-Schwesinger et al., 2020). The expression of murine PLA_2_R1 did not affect any morphological disturbance, showing the intact nephrin with normal foot processes in murine podocytes. Transferring rabbit anti-mPLA_2_R1 antibodies to these mice led to proteinuria and tissue pathological characteristics of MN. Pathological analyses showed the promotion of staining for murine PLA_2_R1 but unaffected staining for murine THSD7A, resembling what is demonstrated in PLA_2_R1-related MN patients. Taken together, this animal model will allow study of underlying PLA_2_R1-specific pathological mechanisms and help to evaluate antigen-specific disease and treatments.

### 2.5 THSD7A-induced mouse model

THSD7A expression is observed in murine podocytes. Mice injected with human serum containing anti-THSD7A antibody and human anti-THSD7A autoantibodies could specifically bind to murine THSD7A on podocyte foot processes, which led to proteinuria, and initiated a pathological change that was typical of MN (Tomas et al., 2016). The mice showed severe albuminuria from 3rd day to 70th day. The mice showed granular changes on GBM subepithelial layer, huIgG-mTHSD7A colocalization and CIC deposition. Immunofluorescence analysis showed C3 accumulation within the subepithelial granular pattern. Similarly, pathological analysis by using an electron microscope strongly demonstrated electron-dense deposits in a limited subepithelial localization and serious foot process effacement surrounding local areas. Subsequently, THSD7A-related MN was put forward in a heterologous animal model (Tomas et al., 2017). Tomas and colleagues established rabbit anti-THSD7A antibodies using co-immunization with combined murine THSD7A and human THSD7A cDNAs. Subsequently, mice were injected by using anti-THSD7A IgG purified from rabbit serum. The mice developed serious nephritic syndrome and increased albumin-to-creatinine ratio during the study period. The mice showed similar pathological characteristics as the above-mentioned THSD7A-related animal model, with the only differences being unknown IgG hypotype and undetected C3.

### 2.6 Anti-dipeptidyl peptidase IV rat model

DPP IV, as a FxlA antigen (gp108) (Natori et al., 1994), is distributed in brush-borders of renal tubules and glomerular capillary loops of renal tissues and gut microvilli. After injecting rabbit anti-DPP IV antibodies into rats, IgG of rabbit was deposited in capillary loops of the glomeruli and generated proteinuria. To identify target antigen, serum DPP IV-depleted rats were used and comparable results were demonstrated. This provided evidence that DPP IV distributed along the capillary wall in the glomeruli had a key effect on proteinuria formation. Compared to the gp330-induced animal model, urinary protein was transient, and here was limited C3 deposition, and faster IgG disappearance (Hunter et al., 1960). Taken together, these studies illuminate the MN pathogenesis model.

## 3 Clinical pathogenesis of IMN

### 3.1 PLA_2_R as a major player in IMN patients

Antibodies are disease specific as they are not observed in other proteinuric nephropathy patients, but are related to MN in several cohorts of patients (Hofstra et al., 2011). Studies have suggested that most MN patients produced an autoimmune response for either PLA_2_R or THSD7A, but not for two antigens (Larsen et al., 2016; Logt et al., 2021), indicating that both antigens are primary target of specific autoimmunity and verifies that PLA_2_R-related MN and THSD7A-related MN are separate disease entities. Several publications have demonstrated that anti-PLA_2_R antibody titre correlate with disease effect and patient outcomes (Brglez et al., 2020; Logt et al., 2021; Nieto-Gañán et al., 2021; Zhang et al., 2021). Low anti-PLA_2_R autoantibody concentrations were used for the diagnosis prediction of spontaneous remission (Jurubiță et al., 2021; Reinhard et al., 2021; Zhang et al., 2021). In addition, reduced concentrations of anti-PLA_2_R antibody could also predict proteinuria remission (Radice et al., 2016) and response to the traditional and new CD20-targeted immunosuppressive treatments (Ruggenenti et al., 2015). By contrast, high concentrations of anti-PLA_2_R antibody at baseline were associated with a reduced probability of spontaneous remission (Jurubiță et al., 2021; Reinhard et al., 2021; Zhang et al., 2021) and were related to progression to nephrotic syndrome in early non-nephrotic proteinuria patients (Gu et al., 2021; Tomas et al., 2021), who showed a high recurrent risk and progressive decline and loss of renal function (Gu et al., 2021; Tomas et al., 2021; Wu et al., 2021; Zhang et al., 2021). Similarly, several previous studies have suggested that anti-THSD7A antibody concentrations could predict disease activity and response to therapy (Hoxha et al., 2017). However, a recent study described a few patients with dual antibody positivity by using renal biopsy staining (Larsen et al., 2016). The remaining IMN patients might either be mediated by autoantibodies against yet unfound antigens or mirror misclassified PLA_2_R-related MN, THSD7A-related MN, or secondary MN. Therefore, the term IMN might no longer be appropriate.

Disease severity and response to drug intervention of MN patients is affected by circulating anti-PLA_2_R autoantibody titre. PLA_2_R1, as a membrane receptor, occurs extracellular region which contains a cystein-rich domain and eight different C-type lectin domains (CTLD1-8) (Xu et al., 2020; Gu et al., 2021). Cystein-rich domain is the dominant epitope of PLA_2_R1 antigen; while site-directed mutagenesis analyses showed CTLD1 and CTLD7 as additional and distinct PLA_2_R1 epitopes that are potential targets of anti-PLA_2_R autoreactivity (Seitz-Polski et al., 2016). Isolated anti-PLA_2_R reactivity against cystein-rich domain are found in younger in patients, with slight urine protein generation, an increase in spontaneous remission rate, and a decrease in risk to ESRD than patients with antibody reactivity against CTLD1 and/or CTLD7 domains suggesting that epitope profiling changes over time and that epitope distributed to CTLD1 and CTLD7 negatively influence disease (Seitz-Polski et al., 2016). These studies demonstrated that anti-PLA_2_R reactivity limited to the cysteine-rich domain was related to initial and milder stages of disease development. Therefore, combined anti-PLA_2_R reactivity and titre could enhance disease activity monitoring and drug therapy response. Whether similar findings could also be useful to THSD7A antigen is unclear.

In the past four decades, two autoantibodies provided the first clear pathological and physiological rationale for MN treatment. Therefore, combined analyses of circulating anti-PLA_2_R and anti-THSD7A autoantibodies as well as albumin concentrations and proteinuria levels in hypoalbuminaemia patients played an important role in understanding disease activity and forming personalized treatment using the common immunosuppressive approach or B cell-targeting monoclonal antibodies (De Vriese et al., 2017; Dong et al., 2020). Indeed, altered antibody concentrations provide a rapid and accurate indication of immune status and an integrated assessment of serum and proteinuria improve treatment decision-making and address diagnostic uncertainties when the proteinuria concentration is consistent with antibody levels. Specifically, persistent proteinuria levels but low antibody concentrations reflect chronic impairment, whereas high antibody concentrations but few obvious clinical symptoms indicate imminent recurrent disease. This integrated strategy to MN enhances accuracy of diagnosis and prognosis, restricts unnecessary intervention to immunosuppressive treatment, optimizes therapy efficacy, and reduces risk or recurrent disease severity in allotransplants (De Vriese et al., 2017). Currently, these principles are addressed in prospective research designed to detect whether treatment titration for autoantibody titre will improve the probability of remission from nephrotic syndrome and inhibit relapse while reducing the risk of treatment adverse effects. The levels of serum albumin and proteinuria remain the most sensitive markers for the prediction of disease severity and outcome in antibody-negative patients.

### 3.2 Activation of complement signalling pathway in IMN patients

The complement role in inducing proteinuria in MN animal models is known, and in human, C5b-9 complexes in urine are a biomarker response to disease reactivity. Although C3 and C5b-9 are universal expressions for biopsy specimens of patients with MN, the underlying pathomechanisms of activation of complement and its relative effects on clinical phenotype after activation of autoimmune system is perplexing (Oto et al., 2021). The typical pathway, such as increased C1q deposition, is related to the cases of secondary MN, rather than idiopathic MN. The mannose-binding protein indicated that alternative or lectin pathway was involved in MN pathogenesis (Borza, 2016; Hayashi et al., 2018). Some idiopathic MN patients occur due to genetic and functional deficiency and an alternate pathway is the functionally activated in these cases (Bally et al., 2016). Although *in vitro* experiments have shown that anti-PLA_2_R affects podocytes, there is no evidence that anti-PLA_2_R alone induced proteinuria *in vivo*. Tomas et al. demonstrated that passive transfer of human anti-THSD7A that expressed THSD7A on podocytes in mice initiated histopathological alternations of MN with proteinuria induction at day 3 despite a lack of activation of complement (Tomas et al., 2016). So far, few findings have revealed how anti-PLA_2_R and anti-THSD7A autoantibodies interplay with the complement system; this link may play a key role in increasing our understanding of the underlying mechanism driving changing proteinuria levels and clinical consequence in this subgroup of patients.

### 3.3 The identification of new antigens in IMN patients

From 2009, a causal antigen was found, when an unprecedented study found PLA_2_R as a target antigen in IMN. This achievement was followed, in 2014, by the discovery of a second antigen, THSD7A in IMN. Since 2014, some studies have reported the identification of several novel minor antigens in the podocytes of MN patients. The first new proteins and antigens were exostosin1/exostosin2 (EXT1/EXT2). EXT1/EXT2 were the most common among the unique proteins in patients with PLA_2_R-negative MN and are present in patients with secondary MN (Sethi et al., 2019; Salvadori and Tsalouchos., 2022). In patients with EXT1/EXT2-positive antigens compared to patients with EXT1/EXT2-negative antigens; nephrotic proteinuria is found more often in patients with membranous lupus nephritis, than in patients with decreasing glomerulosclerosis and renal fibrosis. Patients EXT1/EXT2-negative reach ESRD quickly and frequently (Ravindran et al., 2021). This was followed by identification of neural EGF-like-1 protein (NELL-1) occurred in 5–10% of patients, which is the second most common MN antigen (Sethi et al., 2020; Wang et al., 2021). NELL-1 was much more common in patients with segmental MN (Kudose et al., 2021). The third new antigen, not common but unique, was semaphorin 3B (Sema3B) which presented in pediatric patients (Sethi et al., 2020). Lastly, the latest study reported that protocadherin 7 (PCDH7), which belongs to both IgG4 subclasses, is another new antigen identified in patients with PLA_2_R-negative MN (Sethi, 2021), and is the third most common novel protein, after EXT1/EXT2 and NELL-1, in patients with PLA_2_R-negative MN. Additionally, high temperature recombinant protein A1 (HTRA1), which belongs to both IgG4 subclasses, were identified in some anti-PLA_2_R-negative IMN patients (Sethi et al., 2021). EXT1/EXT2, NELL-1 and Sema3B belong to the IgG1 subtype, similarly, PCDH7 was an anti-PLA_2_R antibody of IgG4 subtype and did not activate complement. Subsequent research is needed to verify the prevalence of these new proteins and putative antigens in MN.

In addition, tumor antigens, thyroglobulin, hepatitis B, hepatitis C and *Helicobacter pylori* antigens and DNA-containing material were identified in subepithelial immune complex deposits in secondary MN patients (Santoro et al., 2017; Tomas et al., 2021). These antigens might be encompassed in GBM due to physico-chemical features, as for cationic albumin. Alternatively, small, circulating, non-precipitating IgG4 complexes including these antigens might deposit in GBM as is found in the model of chronic serum sickness, although there is still no evidence from animal models to demonstrate this hypothesis in humans.

So far, all identified antigens are responsible for up to 90% of IMN patients, therefore, the future search for further minor antigens should not be abandoned (Ahmad and Appel., 2020). Approximately 1% of MN patients have double positivity for anti-THSD7A and anti-PLA_2_R antibodies (Kaya et al., 2021; Lwezaula et al., 2021; Zhang et al., 2021). In biopsy-proven MN patients, anti-THSD7A antibodies were found in 2.8% patients, eight of which were double positive for two antibodies (Zaghrini et al., 2019; Zhang et al., 2019; Zhang et al., 2021). Different antigens may be implicated in various pathogenesis, mode of histologic injury and various clinical phenotype including response to therapy and outcome (Beck, 2017; Sethi et al., 2021).

## 4 Systems biology-Based mechanisms in IMN

Systems biology including genomics, transcriptomics, proteomics, and metabolomics could offer underlying pathomechanisms to illuminate complex and refractory diseases. Genomics, transcriptomics, proteomics, and metabolomics are related to genome (DNA), transcriptome (RNA), proteome (proteins) and metabolome (metabolites), respectively (Zhao and Lin., 2014). High morbidity and mortality of chronic kidney disease (CKD) has emerged as a major public health problem worldwide (Mantovani and Chiara., 2020; Malaki, 2022; Rashid et al., 2022). Multi-omics techniques have been extensive applied to the biomarker discovery, diagnosis and prognosis, drug pharmacological and toxicity evaluation in a variety of diseases including (Cisek et al., 2016; Yang, 2020; Joshi et al., 2021). Although a number of studies of systems biology have been performed on exploring the pathomechanisms of CKD through analyzing DNA, RNA, proteins, metabolites and their molecular and metabolic pathways, the metabolomics in MN is still in its infancy compared with other CKD (Jiang et al., 2013; Cisek et al., 2016; Taherkhani et al., 2019).

### 4.1 The dysbiosis of gut microbiota in IMN patients

Increasing evidence has demonstrated that the dysbiosis of gut microbiota are implicated in CKD (Chen et al., 2019; Feng et al., 2019; Tsay et al., 2019; Chen et al., 2020; Liu et al., 2021; Zhao., 2022). Recently, several studies suggest that the dysbiosis of gut microbiota also contribute to the pathological mechanisms of MN (Kronbichler et al., 2015). The latest study showed that IMN patients had a significantly different α-diversity and β-diversity compared with CKD patients and healthy controls (Zhang et al., 2020). IMN patients presented with increased Fusobacteria and Proteobacteria, but decreased Firmicutes compared to healthy controls. At genus level, *Megasphaera*, *Megamonas*, *Akkermansia*, *Lachnospira*, *Roseburia* and *Fusobacterium* were higher abundant in healthy controls than CKD and IMN (Zhang et al., 2020). Faecal analysis showed the decrease in propionate and butyrate in IMN than healthy controls. Compared with the healthy controls, *Parabacteroides* had elevated abundance in CKD and IMN patients. In addition, *Oscillospira* and *Ruminococcus* had higher abundance in CKD patients than in IMN patients and healthy controls. At the genus level, 10 bacterial taxa had higher abundance in the healthy controls. *Providencia* and *Myroides* had higher abundance in IMN patients (Zhang et al., 2020). Collectively, these findings suggest IMN patients had a significantly different composition and reduced gut microbiota-derived short-chain fatty acids.

Another study showed that *Escherichia-Shigella, Streptococcus*, *Peptostreptococcaceae_incertae_sedis,* and *Enterobacteriaceae_*unclassified were more abundant, while *Lachnospira, Lachnospiraceae_*unclassified*, Clostridium_sensu_stricto_1* and *Veillonella* were less abundant in patients with kidney biopsy-proven MN (Dong. et al., 2020). *Megasphaera* and *Bilophila* were more abundant, whereas *Megamonas, Veillonella, Klebsiella* and *Streptococcus* were less abundant in patients with kidney biopsy-proven immunoglobulin A nephropathy (IgAN) than in those with MN patients (Dong, R. et al., 2020). A negative correlation was shown between *Escherichia-Shigella* and proteinuria, *Bacteroides* and *Klebsiella* presented a positive correlation with MN stage (Dong, R. et al., 2020). In addition, Yu et al. reported MN patients exhibited more severe dysbiosis of gut microbiota than patients with diabetic kidney disease (Yu, W. et al., 2020). A higher number of pathogens in MN were major contributors to microbiome changes in MN. Metabolic pathway analysis showed the increase in interconversion of pentose/glucoronate and membrane transport in correlation to adenosine triphosphate-binding cassette transporters and phosphotransferase system in MN (Yu, W. et al., 2020). Faecal microbiota transplantation (FMT) is applied to treatment of patient with MN and chronic diarrhoea, whose mitigated symptoms and improved renal function (Zhou et al., 2021).

### 4.2 Non-coding RNAs in MN

#### 4.2.1 The dysregulation of long non-coding RNAs in MN

The dysregulation of gene expression through lncRNAs because of epigenetic alternations are increasingly recognized as principal factors in many diseases including kidney diseases (Wang et al., 2021). An earlier publication identified the first dysregulated lncRNAs, X-inactive specific transcript (XIST) and nuclear enriched abundant transcript 1 (NEAT1), whose levels are significantly increased in both tubular epithelial and glomerular cells (Huang et al., 2014). The mouse podocytes treated by lipopolysaccharides led to the stable increase of XIST, but not NEAT1 levels. XIST could be detected in mouse urine, with a highly correlation to disease severity, but not serum in MN. H3K27me3 levels were downregulated in mouse kidney of MN, where also showed decreased H3K27me3 at XIST promoter regions (Huang et al., 2014). The further analysis showed that urinary XIST was significantly increased in the urine of MN patients (Huang et al., 2014). Together, the finding suggests that reduced H3K27me3 at XIST promoter regions resulted in the increased XIST levels in urine. Subsequently, the increase in XIST expression and angiotensin II levels as well as kidney and podocyte damage were observed in kidney tissues of IMN patients (Jin et al., 2019). The upregulated XIST expression induced podocyte apoptosis treated by angiotensin II, while downregulated XIST expression reversed podocyte apoptosis. MiR-217 was negatively regulated by XIST expression. MiR-217 could control Toll-like receptor 4 (TLR4) protein by targeting its 3′-untranslated region. XIST expression modulated TLR4 protein through miR-217 expression and inhibition of XIST expression decreased podocyte apoptosis mediated by angiotensin II via regulating miR-217 (Jin et al., 2019). These findings suggest that the downregulated XIST expression suppressed podocyte apoptosis via miR-217-TLR4 signalling pathway, which could retard IMN. The latest study reported that a total of 327 differentially expressed mRNAs and 48 MN-related immune genes were identified (Li et al., 2021). The mRNAs including FOS and JUN may be implicated in MN through response to immobilization stress and osteoclast differentiation. The mRNA SRY-box transcription factor 4 could contribute to MN through sponging KCNQ1OT1-miR-204-5p interaction (Li et al., 2021). Collectively, these findings suggest that the dysregulation of gene expression of lncRNAs were involved in MN.

#### 4.2.2 The dysregulation of microRNAs in MN

The microRNAs (miRNAs) play key roles in the posttranscriptional gene regulation. Accumulated evidence has demonstrated the dysregulation of miRNAs were involved in CKD (Zhao et al., 2019). Several studies identified the dysregulation of miR-193a and miR-217 in animal models and patients with MN (Li et al., 2019; Zhang et al., 2019). Wilms’ tumor 1 (WT1) was demonstrated as a target gene of miR-193a and WT1 expression were upregulated after miR-193a inhibition (Li et al., 2019). The IMN patients showed the upregulated miR-193a expression and down-regulated WT1 and podocalyxin expression compared to healthy controls (Zhang et al., 2019). Moreover, upregulated miR-193a expression, downregulated WT1 and podocalyxin expression, increased levels of proteinuria/serum creatinine and declined estimated glomerular filtration rate (eGFR) were implicated as prominent markers for poor survival in IMN patients (Zhang et al., 2019). Of note, miR-193a combined with podocalyxin and WT1 presented an optimal effect in differentiating IMN patients from controls and in anticipating survival state. In line with patient’s results, the mRNA level of miR-193a was significantly higher in CBSA-induced MN rats than that in control rats (Li et al., 2019). Inhibition of miR-193a expression ameliorated renal and podocyte injury as well as tissue cell apoptosis in MN rats. Upregulated miR-193a expression was associated with the downregulated expression of podocalyxin, nephrotic syndrome type 1 and Notch1 in MN rats (Li et al., 2019). Inhibition of miR-193a expression by targeting WT1 inhibited kidney function decline and cell apoptosis in renal tissues. In contrast to miR-193a expression, the miR-217 expression was significantly downregulated and tumor necrosis factor ligand superfamily member 11 was significantly upregulated in MN compared with control patients (Rani et al., 2017). The tumor necrosis factor ligand superfamily member 11 was confirmed to be the target gene of miR-217. The upregulated miR-217 expression inhibited tumor necrosis factor ligand superfamily member 11 expression and reduced human podocyte apoptosis (Rani et al., 2017). Similarly, the downregulated miR-217 expression was also observed in kidney tissues of IMN patients compared with normal kidney tissues, and XIST downregulation alleviated podocyte apoptosis via the miR-217-TLR4 pathway in IMN (Jin et al., 2019).

In addition, the miR-186 expression was significantly downregulated while the protein expression of TLR4 and P2×7 was upregulated in renal tissues of MN patients (Sha et al., 2015). *In vitro* experiments showed that TLR4 siRNA led to the upregulated miR-186 expression and miR-186 inhibitor upregulated mRNA and protein expression of P2×7 as well as cleaved-caspase-3 levels in podocytes induced by angiotensin II. The TUNEL-positive cells and caspase-3 activity of podocytes mediated by angiotensin II were downregulated by miR-186 mimic (Sha et al., 2015). The latest study reported that the expression of miR-19b, miR-106a, and miR-17 was significantly downregulated in IMN patients, whereas phosphatase and tensin homolog (PTEN) protein expression was significantly upregulated compared with controls (Wu et al., 2021). The expression of miR-106a and miR-19b was negatively correlated with serum PTEN protein expression, which were positively correlated with serum creatinine, cystatin C, urea, 24 h urine total protein and negatively correlated with albumin and eGFR (Wu et al., 2021). In addition, downregulated miR-500a-5p was identified in kidney tissues of IMN patients, which participated in the regulation of podocyte apoptosis through Circ_0000524/miR-500a-5p/CXCL16 pathway in MN (Sun et al., 2021). Besides renal tissue samples, 326 miRNAs showed a significant difference in peripheral blood lymphocyte cells between MN patients and healthy controls, which included 286 decreased miRNAs and 40 increased miRNAs (Chen et al., 2014). Of note, six novel miRNAs showed differential expression levels between MN patients and healthy controls (Chen et al., 2014). Another study showed that both peripheral blood and urinary miR-192-3p, miR-195-5p, miR-328-5p, and their target genes RAB1A, PPM1A and BRSK1 may be potential markers for MN by taking part in inflammation (Zhou et al., 2019). Collectively, miRNAs provide a potential progression in the pathogenesis of MN and provide a new marker for diagnosis and intervention of MN.

### 4.3 The disorder of proteome in MN

Proteomic biomarkers might improve the management of CKD patients by enabling more accurate and earlier prediction of renal function than was possible with serum creatinine and urinary albumin. Earlier studies from Ghiggeri’s research group identified the podocyte neo-expressed intracellular MN antigens by proteomic approach (Murtas and Ghiggeri., 2016). Specific anti-aldose reductase (AR) and anti-manganese superoxide dismutase (SOD2) IgG4 were detected in serum of MN patients and had high titers of anti-AR and anti-SOD2 IgG4 from the glomeruli of MN kidneys but not other glomerulonephritides (Prunotto et al., 2010). The co-localization of anti-AR and anti-SOD2 with IgG4 and C5b-9 were observed in electron-dense podocyte immune deposits. An increase of SOD2 expression was observed on podocyte plasma membrane after treatment with hydrogen peroxide (Prunotto et al., 2010). Another study demonstrated the existence of three immune proteins including α-enolase, elongation factor 2 and glycyl aminoacyl-tRNA synthetase in glomeruli of patients with membranous glomerulonephritis (Bruschi et al., 2011). The co-localization of α-enolase with IgG4 and C5b-9 in immune-deposits was considered an auto-antigen. Anti-α-enolase IgG4 levels were observed in serum of 131 patients with membranous glomerulonephritis and were elevated in 25% of patients (Bruschi et al., 2011). In addition, further study indicated that IgG4 is the prevalent isotype for antibodies against cytoplasmic antigens of podocytes such as AR, SOD2 and α-enolase. Their levels were higher than in other proteinuric glomerulonephritides and normal controls and were correlated with anti-PLA_2_R levels (Murtas et al., 2012).

Based on the tandem mass spectrometry technique, Sui et al. identified a total of 423 differential proteins in biopsy tissues of MN patients compared to controls. Of these, upregulated 202 proteins and downregulated 221 proteins were associated with immune response activation (Sui et al., 2015). Li et al. identified 249 proteins that were associated with immunization and coagulation and the increased excretion of α-1-antitrypsin and afamin were demonstrated in urine of IMN patients compared with normal controls (Pang et al., 2018). These findings revealed significant role of immunologic mechanism in the development of IMN. Using an animal model of passive Heymann nephritis, Ngai et al. identified Most of altered proteins in urine have functional significance in glomerular trafficking and controlling the glomerular permeability (Ngai et al., 2006).

The deposition of complement is characteristic of MN (Tsai et al., 2019). Traditional renal biopsy assessment is limited to C1q and C3c staining, which showed only limited information and understanding of the pathomechanisms of complement in MN. Ravindran et al. identified high abundance for PLA_2_R and EXT1/EXT2 in corresponding patients of PLA_2_R- and EXT1/EXT2-positive MN that had high abundance of complement proteins including C3, C4, C5, C6, C7, C8 and C9 (Ravindran et al., 2020). C1 was observed in low abundance in EXT1/EXT2-related MN. Complement activation regulators were detected in MN including upregulation of factor H, factor H-related (FHR)-1, FHR-5, clusterin and vitronectin expressions as well as downregulation of FHR-3, FHR-4 and CD59 expressions (Ravindran et al., 2020). IgG4 and IgG1 were the most abundant IgG subclasses in PLA_2_R- and EXT1/EXT2-related MN. These findings conclude that anti-complement agents might be effective in treatment for MN. The identified proteins were related to the pathogenesis of MN and might represent candidate MN markers. These studies underscore the important influence of proteomics in illuminating pathophysiology and biomarker discovery of MN.

### 4.4 The disorder of metabolites in MN

Metabolomic technique has been extensively applied to various CKD (Zhao., 2013; Chen et al., 2016; Chen et al., 2017; Chen et al., 2019; Feng et al., 2019; Miao et al., 2020). PLA_2_R was used for the diagnosis and monitoring of MN, whereas the underlying mechanisms of PLA_2_R-related MN remain unclear. In urine, Kim et al. investigated metabolomic profiles of PLA_2_R-related MN patients with biopsy-proven with those of patients with minimal alteration disease and to healthy controls (Jo et al., 2021). Fumarate was identified as a significant altered metabolite in PLA_2_R-related MN compared with minimal change disease. High fumarate levels in urine could indicate composite outcome of MN. Fumarate hydratase, which could hydrolyze fumarate, colocalized with downregulated podocalyxin expression in glomerulus from patients with PLA_2_R-related MN than in those from healthy controls, patients with non-PLA_2_R-related MN or minimal change disease. Podocytes stimulated with IgG purified from high serum anti-PLA_2_R titre reduced fumarate hydratase expression and increased fumarate levels, which were associated with the changes in the expression of the podocytes phenotypic profile including WT1, zonula occluden-1, Snail, and fibronectin, an elevation in albumin flux across podocyte layer and reactive oxygen species production in podocytes. Fumarate hydratase overexpression retarded these alterations, while fumarate hydratase knockdown showed synergistic effects. Therefore, fumarate could promote alterations in the phenotypic profiling of podocytes after PLA_2_R autoimmunity development. These studies indicated that fumarate could be considered as a promising target for PLA_2_R-related MN treatment.

Membranous glomerulonephritis is one of causes of nephrotic syndrome in adults. Taherkhani and his colleagues identified a panel of metabolites such as allantoic acid, deoxyuridine, oxalic acid, 2-hydroxyglutaric acid lactone, 3,4-dihydroxymandelic acid, α-hydroxybutyric acid, 5α-cholestanone, nicotinamide, epicoprostanol and palmitic acid that associated with nine impaired pathways such as pyrimidine metabolism and NAD salvage in patients with membranous glomerulonephritis (Taherkhani et al., 2018; Taherkhani et al., 2019). Hao et al. identified increased glucose, dimethylamine and trimethylamine levels as well as decreased pyruvate, valine, hippurate, isoleucine, phenylacetylglycine, citrate, tyrosine, 3-methylhistidine and β-hydroxyisovalerate levels in urine of focal segmental glomerulosclerosis compared with healthy controls (Hao et al., 2013).

Park et al. identified increasing urinary glycine levels were related to lower risk of eGFR decline in IgAN patients. Human kidney tubular epithelial cells treated by glycine mitigated inflammatory signaling mediated by tumour necrosis factor-α. This finding suggests that urinary glycine might be a protective biomarker and has a diagnostic and prognostic value for IgAN. Taken together, these findings suggest that disorder of metabolites were implicated in the pathomechanisms of MN and the metabolites could be considered as promising biomarkers for the prediction of MN.

### 4.5 DNA methylation in MN

Histone H3K4 trimethylation (H3K4 me3) that was demonstrated in active euchromatic regions played a key role in podocyte function that actin filaments were high in foot processes of glomeruli. H3K4 me3 showed a pattern of nuclear expression in renal podocytes of MN patients. Overlapping H3K4 me3 and cathepsin L expression were increased in MN patients compared with controls. H3K4 me3 was negatively and positively correlated with synaptopodin and proteinuria levels in MN patients, respectively (Fujino and hasebe., 2016). H3K4 me3 levels were increased in podocytes of mice treated by lipopolysaccharide, accompanied by increased podocyte swelling, creatinine in serum and albumin and cathepsin L in urine as well as downregulated synaptopodin expression. H3K4 me3 expression at cathepsin L promoter was increased in lipopolysaccharide-induced mouse kidneys (Fujino and hasebe., 2016). Treatment with shRNA against mixed-lineage leukemia protein 3 to lipopolysaccharide-treated mice and podocytes cocultured with lipopolysaccharide-induced macrophages mitigated podocyte swelling, increased creatinine levels in serum and albumin levels in urine and upregulated expression of histone H3K4 me3 and cathepsin L, and downregulated synaptopodin expression and upregulated in H3K4 me3 expression at the promoter of cathepsin L (Fujino and hasebe., 2016). These findings suggest that H3K4 me3 upregulation was implicated in podocyte injury and MN pathophysiology. Targeting H3K4 me3 followed by regulating the actin dynamics might be an effective strategy to retard MN outcomes. In addition, Sui et al. reported that 108 genes were significantly different expression in peripheral blood mononuclear cells from MN patients compared with healthy controls (Sui et al., 2014). MN patients showed increased activity of 75 H3K9me3 genes and reduced activity of 33 compared with controls. Five positive genes were selected and quantified (Sui et al., 2014). These findings suggest that H3K9me3 might be a target for epigenetic-associated MN therapy.

In addition, mounting studies have demonstrated that chronic kidney diseases were associated with transforming growth factor-β1 (TGF-β1)/Smad, Wnt/β-catenin and Keap1/Nrf2 signaling pathway (Lan, 2021; Li et al., 2021; Luo et al., 2021; Yu et al., 2022). Whether these signaling pathways are involved in the pathogenesis of MN, recent several publications show that these signaling pathways are implicated in MN (Lee, 2011; Sutariya et al., 2016; Kiewisz et al., 2019; Dong et al., 2021). Therefore, these signaling pathways should be further investigated to find the novel therapy for MN.

## 5 Treatment of traditional Chinese medicine on MN

### 5.1 Treatment of traditional Chinese medicine on patients with MN

Traditional Chinese medicines have been extensively demonstrated to be an important therapy for the CKD treatment (Wang et al., 2018; Geng et al., 2020; Meng et al., 2020; Fang et al., 2021; Cao et al., 2022; He et al., 2022; Liu et al., 2022; Miao et al., 2022). Increasing publications have showed that traditional Chinese medicines exhibited the excellent efficacy in the treatment of patients with MN (Yang et al., 2021; Wang et al., 2022) (Table 1). A previous study has demonstrated that a 77-year-old woman with IMN was intervened with *Astragalus membranaceus* and showed remission (Ahmed et al., 2007). In addition, a multicenter randomized controlled clinical study demonstrated that Shenqi particle were safety and exhibited an efficacy effect for patients with IMN and nephrotic syndrome (Chen et al., 2013). Recent several studies demonstrate that traditional Chinese medicines show the beneficial effect on patients with MN. For instance, treatment with Jian pi qu shi formula improve MN patients who fail to immunosuppressive intervention, and indicate that 80% of patients obtain remission, while no obvious adverse effects are demonstrated after 1 year of follow-up (Shi et al., 2018). Similarly, treatment with Shulifenxiao formula exhibits the efficacy effect for steroid and immunosuppressant-resistant refractory IMN patients (Cui et al., 2021). Therefore, these therapies may be an important option for steroid and general immunosuppressant resistant IMN patients.

**TABLE 1:** Treatment of traditional Chinese medicine on patients and animal models with MN.

Traditional Chinese medicine	Patients or animal models	Effects	Mechanism	Refs
*Astragalus membranaceus*	IMN patients	Decrease in proteinuria and remission	-	Ahmed et al. (2007)
Shenqi particle	Patients with IMN and nephrotic syndrome	efficacy effect	-	Chen et al. (2013)
Jian pi qu shi formula	MN patients who fail to immunosuppressive intervention	80% of patients obtain remission and improvement of proteinuria, serum albumin and cholesterol levels	-	Shi et al. (2018)
Shulifenxiao formula	Steroid and immunosuppressant-resistant refractory IMN patients	81% of patients obtain remission	-	Cui et al. (2021)
Combination TW multiglycosides with prednisone	IMN patients	Similar for both TW multiglycosides and tacrolimus	Regulation of 1485 IMN-related genes and 45 core genes	(Jin et al., 2020; Shi et al., 2022)
Combination of chemical drugs and Yinxingdamo injection	IMN patients	Increase in serum albumin and decrease in proteinuria excretion	-	Yu et al. (2020)
Combination of chemical drugs and Danhong injection	IMN patients	Increase in serum albumin and decrease in proteinuria excretion	-	Yu et al. (2020)
Astragaloside IV	MAC-induced podocyte model	Improvement of complement attack complex-induced podocyte damage	suppressing extracellular regulated protein kinase activity	Zheng et al. (2012)
HP total coumarins	CBSA-induced rats	Amelioration of renal damage and dyslipidaemia	Inhibition of complement activation and PI3K pathways	Wang et al. (2022)
Sanqi oral solution	CBSA-induced rats	Inhibition of proteinuria levels, kidney damage, C3 and IgG depositions	Inhibition of NF-ƙB signaling pathway	Tian et al. (2019)
Wenyang lishui decoction	CBSA-induced rats and IMN patients serum treated-podocytes	Inhibition of proteinuria levels and kidney damage	Regulation of p53 and Bcl-2 in both mRNA and protein expression	Lu et al. (2020)

HP, Hydrangea paniculata; PI3K, phosphoinositide 3-kinase-protein kinase B; TW, Tripterygium wilfordii.

The latest several studies also show that a number of Chinese herbal injections are superior to treatment of chemical drugs alone in improving primary nephrotic syndrome. For example, *Tripterygium wilfordii* shows anti-inflammatory and immunosuppressive activities and improve autoimmune and inflammatory diseases including rheumatoid arthritis, kidney disease and systemic lupus erythematosus. Combining multi-glycosides of *Tripterygium wilfordii* with prednisone is an alternative therapy for IMN. The remission probability is similar for both *Tripterygium wilfordii* multi-glycosides and tacrolimus (Jin et al., 2020). Mechanistically, the latest publication shows that a total of 153 compound-related genes are associated with 1485 IMN-related genes and 45 core genes are overlapped between two categories (Shi et al., 2022). The protein-protein interaction network and MCODE results show that the targets such as tumor protein p53, mitogen-activated protein kinase 8, signal transducer and activator of transcription 3, intercellular adhesion molecule 1, interleukin-4, transforming growth factor β1 and matrix metalloproteinase-1 play critical roles in the treatment of *Tripterygium wilfordii* on IMN (Shi et al., 2022). Enrichment analysis indicates that major pathways are advanced glycation end products, interleukin-17, tumor necrosis factor and Toll-like receptor signaling pathways (Shi et al., 2022). In addition, the combination of chemical drugs and Yinxingdamo injection or Danhong injection showed the best Chinese herbal injections compared with total clinical effectiveness, serum albumin and 24-h urinary protein excretion (Yu, H. et al., 2020). These findings demonstrate that traditional Chinese medicines can effectively ameliorate patients with IMN.

### 5.2 Treatment of traditional Chinese medicine on animal models with MN

The underlying pathogenesis has been investigated and a number of potential molecular mechanisms have been illuminated by using animal models. An earlier study revealed that Astragaloside IV ameliorated complement attack complex-induced podocyte damage through suppressing extracellular regulated protein kinase activity (Zheng et al., 2012). Recent several studies demonstrate that traditional Chinese medicines show a beneficial effect on animal models with MN. For example, treatment with total coumarins of *Hydrangea paniculata* ameliorates renal damage and dyslipidaemia, which are associated with inhibition of complement activation and phosphoinositide 3-kinase-protein kinase B pathways (Wang, W. et al., 2022). After oral administration, pharmacokinetics indicates that plasma 7-hydoxycoumarin and skimmin are main compound or metabolite. This study further shows that 7-hydoxycoumarin and skimmi may suppress interleukin-10 production through blocking phosphoinositide 3-kinase-protein kinase B and nuclear factor ƙB (NF-ƙB) signaling pathways (Wang et al., 2022). Another study shows that treatment with Sanqi oral solution reduces proteinuria levels, blunts kidney damage, suppresses depositions of C3 and IgG, and maintains protein expressions of podocin and synaptopodin in CBSA-induced MN rats, which are associated with NF-ƙB signaling pathway (Tian et al., 2019). In addition, other study shows that wenyang lishui decoction significantly reduces 24 h urine protein level and kidney histological injury in CBSA-induced rats, similarly, *in vitro* experiment shows that wenyang lishui decoction inhibits apoptosis rate in mouse podocytes incubated with RPMI-1640 medium containing 10% serum from patients with IMN, which are associated with decreasing p53 in both mRNA and protein expression and increasing Bcl-2 in both mRNA and protein expression (Lu et al., 2020). These findings reveal that traditional Chinese medicines ameliorate MN through regulating phosphoinositide 3-kinase-protein kinase B and NF-ƙB signaling pathways.

## 6 Future therapies and challenges

Currently, available therapies in MN patients are described in Kidney Disease: Improving Global Outcomes guidelines in 2021. The patients should adopt optimal conservative treatment for at least 6 months or until eGFR decline. Based on proteinuria severity, eGFR change and anti-PLA_2_R antibody levels, patients are eligible for immunosuppressive treatment. Cyclophosphamide, rituximab or a calcineurin inhibitor may be considered based on expected efficacy and side effects. Over the past decade, achievements in the understanding of the pathomechanisms of MN will establish novel therapy perspectives, which have provided an opportunity for the establishment of more specific regimens that were beneficial for MN patients. The discovery of PLA_2_R as the predominant antigen in MN fulfilled an unprecedented breakthrough point in the mechanisms of MN, subsequently, the discovery of serum anti-PLA_2_R antibodies paved the way toward new regimens. Because patients with high serum pathogenic antibodies showed the most severe disease, the removal of antibodies might be beneficial for MN patients. Specific therapies require identification of pathogenic epitopes. Earlier study discovered two mimotopes that mimic conformational epitope recognized by maternal alloantibodies against NEP and immuno-adsorption of maternal serum removed most of the anti-NEP antibodies (Ronco and Debiec., 2017). Currently, a small pilot study of anti-PLA_2_R antibodies immune adsorption is used for establishment of the feasibility of rendering patients seronegative for anti-PLA_2_R antibodies in lack of immunosuppression (Hamilton et al., 2018). Removal of serum anti-PLA_2_R antibodies by immune adsorption ameliorated disease severity, but it could not cure the disease. It will remain necessary to block antibody production. Similarly, the study of THSD7A antigen/antibody is also considered.

If IgG4 antibodies lead to the podocyte damage by binding to PLA_2_R, independent from complement activation, it would provide feasibility to establish therapies by small peptides that would intervene in the binding of the antibodies to PLA_2_R antigen. However, substantial evidence showed that complement was activated and C5b–9 assembly caused podocyte damage. The coexistence of IgG1 in immune complex deposits could mediate complement activation. However, most IMN patients showed very low or undetectable levels of complement C1q in immune complex deposits compared with secondary MN patients. Therefore, these findings indicated that lectin signaling pathway might participate in complement activation and C5b–9 complex formation. Indeed, mannose-binding lectin signaling pathway was found in the glomeruli of most IMN patients. The pathomechanisms of C5b–9 complex formation provides an opportunity to develop targeted therapies through complement-specific antibodies.
